# Lung Function Assessment in Pediatric Asthma: Selecting the Optimal Tests for Clinical and Research Applications

**DOI:** 10.3390/children12081073

**Published:** 2025-08-15

**Authors:** Giulia Michela Pellegrino, Alessandro Gobbi, Marco Fantini, Riccardo Pellegrino, Giuseppe Francesco Sferrazza Papa

**Affiliations:** 1 Dipartimento di Scienze Neuroriabilitative, Casa di Cura Igea, 20144 Milano, Italy; g.pellegrino@casadicuraigea.it; 2 Dipartimento di Elettronica, Informazione e Bioingegneria, Politecnico di Milano, 20133 Milano, Italy; a.gobbi@restech.it; 3 Restech Srl, 20124 Milano, Italy; 4 Otolaryngology Department, Ospedale Koelliker, 10134 Torino, Italy; marco.fantini@gruppokoelliker.it; 5 Centro Medico Pneumologico Torino, Ospedale Koelliker, 10134 Torino, Italy; info@pneumotorino.it

**Keywords:** spirometry, forced oscillation technique, interrupter technique, bronchodilator and bronchoconstrictor challenges, topographical and temporal ventilation heterogeneities, vocal cord dysfunction, dyspnea

## Abstract

Recent documents from leading international pediatric respiratory societies have strongly encouraged the use of lung function tests in clinical practice and research. These tests can explore ventilatory function across its volumetric and temporal domains, providing information on the intrapulmonary location and extent of damage caused by respiratory diseases. The choice of which test to use in each case to investigate presenting respiratory symptoms depends on the patient’s symptoms and the diagnostic–therapeutic phase being addresse d. In the most common and representative chronic pediatric condition—bronchial asthma—lung function tests play an especially important role due to the disease’s complexity and the fluctuating nature of airway obstruction. This review aims to examine the potential of various lung function tests in asthma, helping clinicians and researchers to optimize diagnosis and follow-up with the most appropriate methodology. While spirometry and flow resistance measurements using the interrupter technique have historically been the cornerstones of diagnosis and clinical monitoring in childhood asthma, the advent of new technologies—such as multiple breath nitrogen washout (MBNW) and the forced oscillation technique (FOT)—is opening up the door to a more nuanced view of the disease. These tools allow for an evaluation of asthma as a structurally complex and topographically and temporally disorganized condition. FOT, in particular, facilitates measurement acceptability in less cooperative subjects, both in respiratory physiology labs and even at the patient’s home.

## 1. Introduction

In recent decades, pediatric respiratory medicine has placed growing emphasis on lung function testing. Major international respiratory medicine societies [1,2,3,4,5] have published guidelines for evaluating the functional damage of respiratory diseases, standardizing and structuring tests for shared interpretation. This progress was made possible by the development of reference normative values for parameters by age, height, and sex [6,7,8,9,10,11].

As in adults, pediatric tests indicate the presence and extent of functional damage to the lungs, but not necessarily the underlying mechanisms [11]. For instance, a reduction in FEV_1_ in an asthmatic child indicates airway obstruction, whereas in cystic fibrosis, the same reduction reflects the compression of ectatic airways during forced expiration. In neuromuscular disorders, it represents the loss of respiratory muscle strength and endurance. As noted in the guidelines, combining different tests helps to detect typical functional patterns of various pediatric respiratory diseases and to guide appropriate therapy [1,2,3,4,5].

Starting from the concept that respiratory diseases are inherently complex and characterized by a variety of functional patterns, this review aims to assess the ability of currently available lung function tests to explore and identify the structural and dynamic components of the most common pediatric disease: bronchial asthma [12,13]. A deeper understanding of the mechanisms and their in vivo interactions causing asthma, through lung function testing, is crucial for both clinical care and research. As irreversible changes in lung function begin in infancy, confirming the presence of the disease would be extremely useful to treat the illness before it becomes too severe and out of control.

## 2. Clinical Applications and Discussion

### 2.1. Lung Function Evaluation

The most frequently used tests in pediatric clinics or pulmonary function labs for the initial evaluation of suspected bronchial asthma are spirometry, flow resistance measurement using the interrupter technique, and resistance estimation through respiratory impedance [1]. This first diagnostic step is crucial to confirm or exclude airway obstruction under baseline conditions. For instance, detecting an obstructive ventilatory pattern can immediately support the hypothesis of asthma based on clinical history and symptoms, thus allowing for the prompt initiation of appropriate pharmacological treatment [14]. On the other hand, if the airways appear normally patent, the next step is to select further diagnostic approaches to determine whether the patient’s or their parents’ reported symptoms are consistent with asthma.

Spirometry is traditionally the test that best identifies either normal patterns or obstructive functional damage [11]. Based on fluid dynamics principles [15], it can reveal deviations from the expected ventilatory patterns [11]. However, the test requires considerable patient cooperation during forced expiratory maneuvers from full lung capacity, which often makes the results unreliable and poorly repeatable, especially in preschool or early school-age children [16,17].

**Figure 1:**
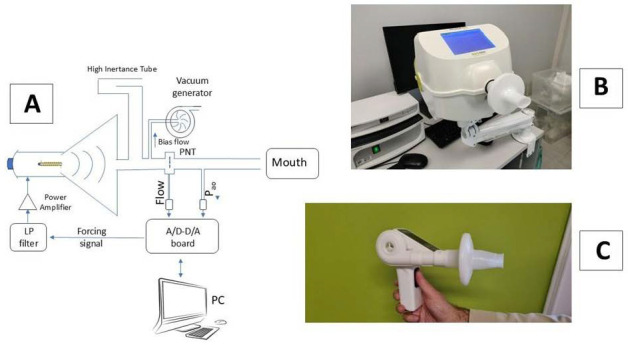
In Panel (**A**), a schematic diagram of the oscillometer is shown, with the loudspeaker on the left generating the desired oscillatory frequencies toward the patient’s mouth. Respiratory flow and mouth pressure are recorded by a pneumotachograph (PNT) and a pressure transducer (Pao), respectively. A bias flow prevents rebreathing. A high-impedance tube forces the oscillatory signal to remain within the measurement system. Pressure and flow measurements at the mouth during oscillations automatically allow for an estimation of the component in the phase with flow (resistance) and the component in the phase with volume (reactance). Panels (**B**,**C**) show examples of commercially available devices used for laboratory testing and home monitoring, respectively.

In some labs, the interrupter technique is used instead, which can assess airway function during quiet breathing with good accuracy [1,18,19], although day-to-day variability tends to be high [20]. The use of the forced oscillation technique (FOT) [21,22,23,24] has opened up new avenues in this field due to its high acceptability, minimal cooperation requirement, and its ability to evaluate lung function during quiet breathing. In pediatric practice, a single low-frequency oscillatory signal (5–8 Hz) is used to facilitate the measurement of resistance (R) and reactance (X) components of respiratory impedance (I) [22,23,24]. R reflects airway patency; an increase suggests airway obstruction. Conversely, X represents the system’s ability to absorb and transmit the oscillatory signal to the peripheral airways and lung parenchyma. A negative X at low frequency in asthma is consistent with ventilation heterogeneity as a result of some airways being so obstructed, particularly in the more peripheral areas, such as better-ventilated regions, that oscillatory flow is redirected to [22,23,24]. Measuring X is of great importance in the field of asthma, as the heterogeneous obstruction of airways within the lungs represents the most typical characteristic of bronchial asthma. This heterogeneity is associated with disease severity, clinical instability, and dyspnea [25,26,27,28,29]. In Figure 1, a schematic diagram of the system is shown (Panel A), along with two commercial systems used in the laboratory (Panel B) and at home (Panel C). Recent guidelines in the field [23] have produced a state-of-the-art summary of FOT measurements in clinical practice in both adults and children, providing standards for device manufacturers, measurement laboratories, and healthcare operators. They also define measurement procedures involving patient–operator interaction, data acquisition duration, criteria for measurement acceptability, and interpretation of the results.

Predicted normal values for FOT are available across pediatric age groups, making the test widely applicable in both clinical and research contexts [8,30]. However, more studies are needed to expand these reference datasets in terms of population size, age range, sex, and ethnicity, especially in preschool children.

Another technique that assesses ventilation heterogeneity in asthma is the multiple breath nitrogen washout (MBNW) [1,4,31,32,33]. This test measures the slope of nitrogen’s phase III during quiet breathing in 100% oxygen. In asthma, the increase in the slope is a marker of uneven ventilation distribution. The Lung Clearance Index (LCI) quantifies the number of breaths required to reduce the nitrogen concentration below a specific threshold. High LCI values indicate ventilation heterogeneity, mainly within the convective-dependent regions of the airways. The test is non-invasive and generally well tolerated by children. However, due to the complexity of the text, its cost, and use limited to the pulmonary function test lab, its clinical application is at the moment quite limited.

In the presence of airway obstruction, as occurs in asthma, all the aforementioned tests are capable of estimating and identifying the event, but not necessarily in the same way, in terms of its nature, severity, or topographical location, given the respiratory system’s complexity. For example, a reduction in forced expiratory flow measured by spirometry indicates airway diameter narrowing, but it also reflects airway wall compliance downstream from the flow-limiting segment and changes in airway caliber caused by the deep inhalation preceding the forced maneuver [15]. Increases in the slope of nitrogen’s phase III and in LCI via MBNW signal heterogeneous bronchoconstriction, particularly in the lung’s more peripheral airways [1,4,31,32,33]. Increases in airflow resistance measured by the interrupter technique or in R measured by FOT are robust indicators of airway obstruction [1,18,19,20,21,22,23,24]. However, due to the respiratory system’s non-uniform response to bronchoconstrictor stimuli, the simultaneous measurement of X and R with FOT allows for the estimation of not only the magnitude of airway obstruction but more importantly of its heterogeneous distribution throughout the lungs; this is a major contributor to bronchial tone instability, disease severity, and dyspnea [25,26,27,28,29].

Other tests, such as the use of FeNO (Fractional exhaled Nitric Oxide), blood IgE, and eosinophils, either as standalone or combined measures, are also considered in the assessment of the disease, both in the diagnostic phase and during follow-up. However, their application is limited to patients with allergic asthma, and clinicians must be aware that some values deemed pathological may not actually be related to bronchial asthma. The gold standard for the diagnosis of bronchial asthma remains the fluctuation in the airway caliber rather than biological markers [14,34].

### 2.2. Bronchomotor Responses in Asthma

Historically, and in line with international guidelines [1,2,3,6,14], the bronchodilator response test is the most widely used in diagnosing bronchial asthma. This is based on the principle that responses exceeding the natural variability limits are consistent with the presence of disease. This test is also preferred due to its feasibility in any clinical setting and the excellent safety profile of the challenge. Bronchodilator reversibility can be assessed using spirometry or respiratory resistance or impedance, as mentioned above, with the known limitations of acceptability and data quality, especially for spirometry.

However, what remains uncertain and not fully standardized is the statistical threshold above which a positive response is considered diagnostic of asthma [6,11]. Clearly, a very large response is highly suggestive of asthma and supports the need for appropriate drug treatment. Borderline or just-above-threshold responses may reflect the effect of the drug on the airways [11] but not necessarily indicate the presence of asthma where additional confirmatory tests may be required.

When it comes to bronchoconstrictor challenges in children, the tests are typically limited to spirometry and FOT. While spirometry presents challenges in ensuring data quality due to the level of cooperation required from the child, FOT is far less affected by this issue, as it analyzes respiratory function during tidal (quiet) breathing with minimal cooperation and optimal acceptability for the patients.

**Figure 2:**
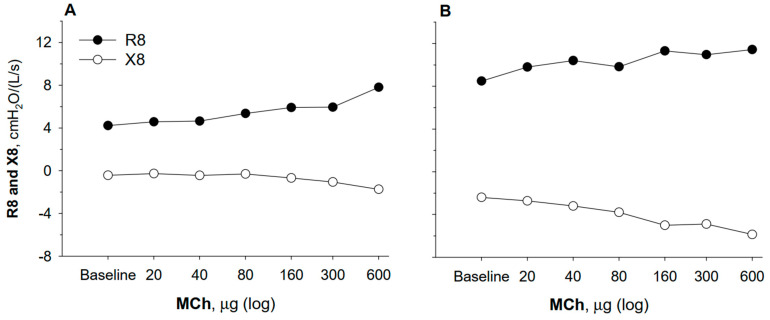
Two typical examples of changes in inspiratory resistance and reactance at 8 Hz (R8 and X8) in two children exposed to methacholine challenge. Dose of the constrictor agent is reported in the horizontal axis. In Panel (**A**), the decrease in X8 is remarkably less than the increase in R8, whereas the opposite is true for the case shown in Panel (**B**). The latter is consistent with large ventilation heterogeneities occurring with airway narrowing.

As mentioned earlier, FOT can assess two different and independent components of respiratory I, R and X, which can reveal different and not necessarily synchronous patterns during bronchoconstrictor response. A drop in reactance (X) during bronchoconstrictor challenge suggests the onset of ventilation heterogeneity—a shift in airflow from obstructed regions to better-ventilated ones [22,23,24]. For example, in the case reported in Figure 2, the inhalation of methacholine in two children caused an increase in airway resistance (indicating airway narrowing) in both of the children but a dominant fall in reactance in the later phases of the test in the subject of Panel B compared to that of Panel A. Based on the assumption that ventilation heterogeneity is a hallmark of asthma for disease severity, clinical instability, and symptoms [25,26,27,28,29], partitioning the two components of I with FOT might be of great help to identify patients prone to developing ventilation heterogeneity, and thus at greater risk of unstable disease.

**Figure 3:**
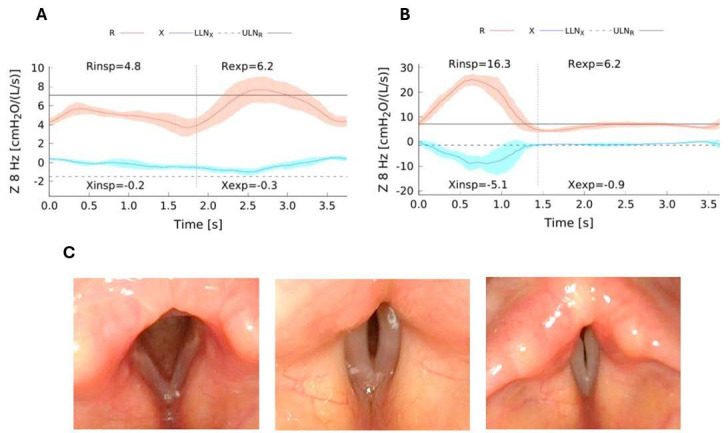
Panel (**A**) Respiratory impedance (Z) and its resistive (R) and reactance components (X) at 8 Hz during tidal inspiration (Rinsp and Xinsp, respectively) and expiration (Rexp and Xexp, respectively) at baseline conditions. Lower and upper limits of normality for R and X are shown by horizontal continuous and dashed lines, respectively. Panel (**B**) Rinsp and Xinsp on tidal inspiration (Risnp and Xinsp, respectively) and expiration (Rexp and Xexpp, respectively) at the time of inspiratory dyspnea appearance during the bronchial challenge. Note the remarkable increase in Rinsp and decrease in Xinsp well beyond their relevant limits of normality, in stark contrast with Rexp and Xexp. Panel (**C**) Laryngological evaluation: normally abducted vocal folds (**left**); vocal cords during phonation (**mid**); paradoxical adduction during inspiration (**right**).

In Figure 3, we report a case of inspiratory dyspnea in a 12-year-old boy during a methacholine (MCh) challenge for suspected asthma. He had a persistent cough for over a year and was treated for at least one year with inhaled steroids and long-acting bronchodilators with no clinical benefits at all. At baseline, the boy showed no dyspnea, and inspiratory R and X were within the normal Z-score limits [8] (Figure 2A). MCh inhalation from 20 mcg to 160 mcg during tidal breathing caused no symptom or mechanical changes. However, the next dose triggered sudden, acute cough followed by dyspnea, stridor, and jugular retraction during inspiration. At that point, inspiratory R significantly increased and X dropped well below the normal limits (Figure 2B), peaking during mid-tidal inspiration. In the next expiratory phase, R returned to normal, and X increased. This pattern was consistent with vocal cord dysfunction. Laryngologic evaluation confirmed normal anatomy and motion, but video laryngostroboscopy revealed paradoxical vocal fold movement—an adductory tendency during inspiration (Figure 3C). No other test could have so clearly and confidently identified this laryngeal reaction (rather than bronchospasm) as the cause of the acute dyspnea during the methacholine challenge. These two examples really document the potential of FOT to explore the complexity of the bronchoconstrictor response in asthma, though they are far from being generalized.

### 2.3. Temporal Variability in Asthma

According to the current GINA 2025 guidelines [14], home monitoring of lung function in asthma can be performed using peak expiratory flow (PEF) or forced expiratory volume in 1 s (FEV1) measurements in the morning and evening. These tests are based on the assumption that PEF or FEV1 are functional markers of asthma, and that measuring them twice daily over a couple of weeks can reveal one of the most characteristic features of asthma—daily variability.

Although there is full agreement in the scientific community that temporal variability is a hallmark of asthma and can be very helpful diagnostically, these tests are rarely used in clinical practice, despite the low cost of the equipment. This is mainly due to the difficulty in obtaining reliable and repeatable results and the uncertainty surrounding its physiological interpretation [11].

In contrast, using the forced oscillation technique (FOT) to measure variability in lung function in asthma appears much more promising, as recently demonstrated [35,36,37,38,39]. This is due to its ease of execution, patient acceptability, result accuracy, and its statistical superiority compared to PEF [39]. In healthy adults, the normal variation coefficient for resistance (CV_R_) is around 0.10. A similar value has recently been confirmed in children [35].

**Figure 4:**
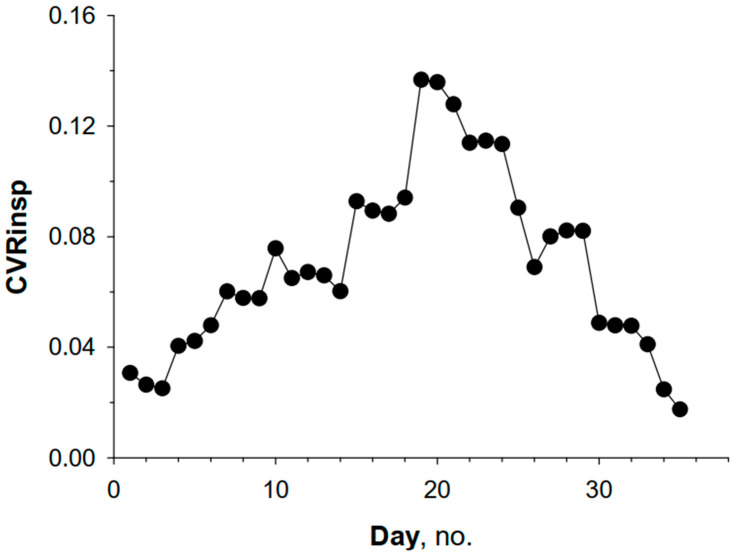
Coefficient of variation in inspiratory resistance (CVRinsp) at 8 Hz measured in the morning for 32 days in an 8-year-old boy. Note a slight initial increase in the CV that well exceeded the expected threshold of 0.10 after about a couple of weeks. This was associated with dyspnea and wheezing. Note the gradual return of CV to normal values after treatment with a combination of inhaled steroid and bronchodilator agent for 1 week.

In the example shown in Figure 4, the values of the coefficient of the variation in inspiratory resistance (CVRinsp) gradually increase beyond the expected normal range, thus suggesting significant instability of the bronchial tone. In such cases, asthma treatment is considered effective if R returns to within normal limits, both in terms of absolute values and in their temporal variability (CV). Definitive reference values for the variability coefficient of R in children will be available soon.

## 3. Potential of FOT in Research

Next, we present several aspects of FOT’s ability to investigate the complex airway dynamics during respiration, which have so far been studied in adults but are also highly relevant in bronchial asthma. Investigating these mechanisms—which play an important role in bronchospasm—in pediatric populations could certainly open up new avenues in understanding the pathophysiology of asthma from an early age.

For example, in healthy individuals exposed to a bronchial challenge, airway resistance decreases after taking a deep breath and gradually returns to baseline within 1–2 min. In asthma, by contrast, this bronchodilatory effect is diminished, and airway re-narrowing occurs more rapidly [39,40,41]. The inability of the airways to distend theoretically reflects the presence of static remodeling mechanisms that prevent the expected dilation with changes in lung volume [39]. Based on the increased rate of reconstriction—estimated from the slope of the relationship between airflow resistance and time after the deep breath—the reduced bronchodilatory effect reflects the complexity of the respiratory system, in which the high shortening velocity of the smooth muscle plays a dominant role in asthma [41,42], beyond mere structural inextensibility caused by static remodeling. Assessing whether exaggerated airway smooth muscle shortening velocity is already present in childhood asthma will certainly stimulate pharmacological research in this direction and make it possible to test drug effects in vivo in individual patients, something that has not been achieved so far.

In a subgroup of asthmatic patients, taking a deep breath is associated with severe dyspnea episodes, as shown by increased airway resistance and reduced forced expiratory flows [43]. In such cases, FOT significantly contributes to diagnosing deep breath-induced obstruction and supports the pediatric use of bronchoactive inhalation therapy via tidal breathing with a spacer rather than deep breathing.

In a recent study on adults undergoing methacholine-induced bronchoconstriction, the complexity of bronchial tone regulation in relation to lung volume was demonstrated [44]. As tidal volume increases, the airways tend to bronchodilate, as expected based on the known physical relationship between airway diameter and lung volume [45]. However, this occurs significantly more when the inspiration starts from a higher lung volume compared to a lower one. The practical implications of these results, if confirmed in both adults and children, suggest the possibility of studying specific breathing patterns that could help asthmatic patients relieve dyspnea during natural bronchospasm.

To our knowledge, no studies to date have investigated the use of FOT to assess bronchial tone variability across different asthma phenotypes and endotypes in adults nor in children.

## 4. Conclusions and Future Directions

The content presented above demonstrates the immense potential of lung function tests in evaluating asthma and underscores the importance of using these tools to effectively answer the clinician’s or researcher’s specific questions regarding the severity and nature of the disease in each individual case. However, not all tests are created equal, as each explores different aspects of lung function.

Given the anatomical, topographical, and temporal complexity of asthma, it is essential to evaluate airway function either comprehensively or selectively, depending on the clinical or research question being addressed. Spirometry, for example, is grounded in the principles of fluid dynamics, which gives it strong and robust overall results. However, this test cannot decompose the individual mechanisms contributing to airflow limitation—such as lung elastic recoil, upstream resistance before the choke point, and downstream airway collapsibility—since these are deeply interconnected. Additionally, poor cooperation from pediatric patients may reduce data quality and hinder accurate interpretation.

Looking ahead, there are exciting opportunities to evaluate lung function in asthmatic children using innovative techniques such as FOT, both in research and in routine clinical practice when a near-definitive diagnosis is required or when the goal is to identify and quantify the many mechanisms causing airflow limitation in pediatric asthma. The technique is indeed capable of exploring the many functional facets of bronchial asthma that go beyond the simple measurement of respiratory resistance, including the study of ventilatory function in its spatial and temporal dimensions, which characterize the disease’s specific nature. In this sense, home FOT monitoring effectively represents what the current GINA guidelines define as the *gold standard* for evaluating airway caliber variability in asthma [14]. Given its ease of use in children and its enormous exploratory potential, FOT could truly transform the clinical approach to the disease in the years to come, for diagnosis, severity monitoring and control, therapy optimization, and, certainly, scientific research into the complexity of airway obstruction mechanisms that emerge at different stages of a child’s development. Finally, it is important to highlight that these concepts are strongly supported by studies confirming the feasibility and acceptability of FOT in pediatric medicine [35]. More importantly, these studies also demonstrate FOT’s high levels of sensitivity, specificity, and accuracy in diagnosing disease, assessing asthma control, and evaluating severity, both at home and in the emergency department in school-aged asthma patients [35,46]. Currently, however, the use of FOT remains relatively limited in pediatric respiratory physiology labs and for home use. Equipment costs, the complexity of the underlying physical–mathematical concepts, limited educational resources, and the need for further scientific studies are key limiting factors in the widespread adoption of this technology in our professional and scientific community. Future research studies, however, will be necessary to reinforce the concept that childhood asthma is a complex disease and must therefore be evaluated using tools capable of capturing the dynamic mechanisms of the disease within lungs that are continuously changing anatomically and biologically as the child grows. Only in this way will it be possible to develop new dynamic measurement standards across age groups, sexes, and ethnicities.

In light of recently proposed eHealth technologies [47,48,49,50] for the home monitoring of children with bronchial asthma in their everyday environments—using various systems such as monitors, wearable devices/audio–visual sensors, virtual reality technologies, environmental condition monitoring, digital educational platforms, remote medical assistance, and artificial intelligence (AI)—home-based FOT monitoring appears to take on a crucial role. It serves as a vital link between what international guidelines currently define as the gold standard for asthma monitoring [14,30], real-life patient evaluation, and future AI-integrated medical care.
